# Study of Leptin and Adiponectin as Disease Markers in Subjects with Obstructive Sleep Apnea

**DOI:** 10.1155/2014/706314

**Published:** 2014-05-29

**Authors:** Sana Al Mutairi, Olusegun A. Mojiminiyi, Alia Al Alawi, Tahani Al Rammah, Nabila Abdella

**Affiliations:** ^1^Department of Medicine, Faculty of Medicine, Kuwait University, P.O. Box 24923, Safat, 13110 Kuwait City, Kuwait; ^2^Department of Pathology, Faculty of Medicine, Kuwait University, P.O. Box 24923, Safat, 13110 Kuwait City, Kuwait; ^3^Department of Medicine, Al-Amiri Hospital, Ministry of Health, P.O. Box 4077, Safat, 13041 Kuwait City, Kuwait

## Abstract

*Background*. Published studies showed conflicting results of the associations between adiponectin and leptin levels and obstructive sleep apnoea (OSA). In obese patients, plasma leptin is elevated and adiponectin is decreased, and we postulate that these adipokines could be potential markers of clinical and metabolic perturbations in patients with OSA. *Methods*. 147 patients with suspected OSA had polysomnography to determine the Respiratory Disturbance Index (RDI). We measured fasting plasma glucose (FPG), fasting serum insulin, plasma leptin, adiponectin, and full lipid profile. Patients were classified on the basis of the RDI, degree of adiposity, and insulin resistance (IR) (homeostasis model assessment of insulin resistance (HOMAIR)). *Results*. 28.6% of subjects had normal polysomnography, 34.8% had mild OSA, 19.6% had moderate OSA, and 17% had severe OSA. Obesity was more prevalent in subjects with moderate-severe OSA (47%). Adiponectin decreased significantly (*P* = 0.041) with increasing severity of OSA. Though BMI was significantly higher in subjects with severe OSA, paradoxically, leptin was lowest in those subjects independent of gender dimorphism. *Conclusions*. Adiponectin is an independent marker of disease severity in patients with OSA. The paradoxical decrease in circulating leptin, which suggests impaired secretion, deserves further studies as a potential marker of severe OSA.

## 1. Introduction 

Apnea or hypopnea can occur due to partial or complete collapse of the airway during sleep resulting in the syndrome of obstructive sleep apnea (OSA). Factors that increase the risk for OSA include male sex [1–3], obesity [4, 5], age [6], and race [7]. Several studies have shown that obesity and, in particular, central obesity are the strongest risk factors for OSA [8–11]. Chronic OSA can result in mitochondrial dysfunction resulting from tissue hypoxia, increased counter-regulatory hormones, and altered adipocytokine patterns which contribute to the reported strong associations of OSA with clinical and metabolic perturbations that increase the risk of Type 2 diabetes mellitus and cardiovascular disease [12, 13].

However, although the hypothesis that obesity-related factors are the links between the clinical and metabolic manifestations of OSA is an attractive one, the relationship between metabolic disorders and OSA is complex and multidirectional. This complex relationship is due in part to the fact that obesity could be a cause, consequence, or confounding factor of OSA [14]. Furthermore, leptin and adiponectin, the major adipocytokines through which obesity exerts its clinical and metabolic effects, have diametrical pathophysiological actions, whereas leptin increases with the degree of obesity and insulin resistance and in response to hypoxia [15], adiponectin increases with continuous positive airway pressure (CPAP) therapy [16] and is inversely associated with the degree of obesity and insulin resistance [15, 17]. The discordant reports in the literature confirm the complexity of the associations between OSA, obesity, and adipokines [15–20]. Although studies have examined the associations of either leptin or adiponectin with OSA, there are few studies [21–23] that have evaluated the simultaneous use of these adipokines in patients with OSA given their diametrically opposite metabolic effects. These association studies were focused on evaluating if independent associations exist between OSA and/or obesity and circulating adipokines and only the study of Tokuda et al. [21] evaluated circulating leptin and adiponectin in relation to OSA severity. The rationale for our study is based on the fact that, in patients with mild, moderate, or severe OSA, the adipokines could be used as disease markers that reflect the balance between the degree of overproduction and/or resistance to leptin versus the degree of underproduction of adiponectin. Therefore, this study explores the association of these adipokines with the metabolic parameters and clinical and polysomnographic findings in patients with varying degrees of OSA.

## 2. Material and Methods

### 2.1. Patients and Clinical Features

Patients referred to the Sleep Laboratory at the Al Amiri Hospital, Kuwait, because of suspected sleep apnea were invited to participate in the study. 147 (68 M, 79 F) patients with mean (SD) age of 52 (16) years gave informed consent to participate in the study.

The study protocol and procedures were approved by the local ethics committee and, in accordance with the ethical standards of the Helsinki declaration, all participants gave informed consent for their participation. After providing informed consent, fasting blood samples were collected from all patients. Exclusion criteria were refusal to take part in the study, ingestion of any medication that may affect lipid or insulin levels at the time of the study, recent (within the previous six months) hospitalization or visit to the emergency room, intercurrent illness (including cardiovascular disease) or clinically evident infections, overt clinical or laboratory evidence of connective tissue diseases, or hemoglobinopathies.

The subjects were interviewed by a trained nurse who completed a questionnaire on age, smoking status, symptoms of OSA, and an evaluation of daytime dozing using Epworth Sleeping Scale (ESS). The questionnaire also includes past medical history of hypertension and/or antihypertensive medication, cardiovascular diseases, pulmonary diseases mainly chronic obstructive airways disease (COPD), diabetes mellitus, peptic ulcer disease, sinusitis, thyroid diseases, and any other diseases a patient might had encountered in the past. Moreover, it covers family history of similar or related disorders, for example, parasomnias, periodic limb movements, and others.

Patients were diagnosed as having sleep apnea clinically if they were complaining of snoring, excess daytime somnolence, witnessed apnea by their partners or roommate or relatives in presence or absence of a diagnosis of systemic hypertension, loss of libido, or decline in cognitive capabilities. All subjects underwent overnight polysomnography (PSG) with determination of the apnea, hypopnea index (AHI), and the Respiratory Disturbance Index (RDI). The PSG, considered the gold standard diagnostic test of OSA [24, 25], was carried out using the Jaeger Somno Star Pro Polysomnography Sleep Diagnostic System version v4.2.7.4. Diagnosis of OSA was based on the criteria of the American Academy of Sleep Medicine [26].

All subjects had assessment of oropharyngeal crowding and anthropometric measurements taken by trained observers. The oropharyngeal crowding was assessed and scored based on Mallampati's technique for evaluating the oropharynx [27]. All anthropometric measurements were made with the participant wearing light clothes without shoes. Height (to the nearest 0.1 cm) was determined by use of a stadiometer, and weight (to the nearest 0.1 kg) was determined by use of a standardized standing beam balance. The body mass index (BMI) was calculated according to the formula: weight in kilograms divided by the square of the height in meters. Patients with BMI > 30 kg/m^2^ are classified as obese and those with BMI < 25 are classified as normal. Those with BMI > 25 or <30 are classified as overweight. Waist circumference was measured midway between the lowest rib and the iliac crest with the subject standing at the end of gentle expiration, and the hip circumference was measured at the level of the widest diameter around the gluteal protuberance.

Systolic blood pressure (SBP) and diastolic blood pressure (DBP) were measured using a mercury column sphygmomanometer and a cuff suitable to the subject's arm circumference. Blood pressure was measured twice (30 minutes apart) in each patient after resting for at least five minutes, and the average of the measurements was recorded. Hypertension was defined as active treatment with any antihypertensive agent or mean SBP ≥ 140 mm Hg or DBP ≥ 90 mm Hg [28].

### 2.2. Laboratory Methods

#### 2.2.1. Adipokines

Fasting plasma adiponectin (Linco Research, St. Charles Missouri, USA) and leptin (Diagnostics Systems Laboratories, Webster, TX, USA) were measured using commercially available enzyme-linked immunoassay (ELISA) kits. Leptin: adiponectin ratio was calculated. The inter- and intra-assay coefficients of variation on pooled plasma specimens were as previously reported [29].

#### 2.2.2. Other Assays

Fasting plasma glucose (FPG), total cholesterol (TC), triglycerides (TG), high density lipoprotein cholesterol (HDL-C), and creatinine were analyzed on an automated analyzer (Beckman DXC, Beckman Corporation, Brea, CA, USA). The low density lipoprotein cholesterol (LDL-C) was calculated using the Friedewald formula [30].

Fasting serum insulin was determined by an ELISA (DSL-10-1600 ACTIVE, Diagnostics Systems Laboratories, TX, USA). Insulin resistance was calculated using the homeostasis model assessment (HOMA-R) formula using a calculator downloaded from https://www.dtu.ox.ac.uk/homacalculator/download.php (Diabetes Trials Unit, Oxford, URL. HOMA Calculator v2.2. https://www.dtu.ox.ac.uk/homacalculator/download.php (accessed January 2012)). The HOMA calculator also gives estimates of steady state beta cell function (%B) and insulin sensitivity (%S). The lower limit of the top quintile of HOMA-R distribution (i.e., 2.0) in apparently healthy nonobese (BMI < 25 kg/m^2^) Kuwaiti subjects was used as the cutoff for defining insulin resistance [29].

#### 2.2.3. Statistical Analyses

Statistical Package for the Social Sciences (SPSS) version 17.0 for windows software (SPSS Inc., Chicago, IL) was used for statistical analysis and *P* < 0.05 was considered statistically significant for all analyses. Data are presented as mean and standard deviation. Comparisons of continuous variables between two groups were made with the Mann-Whitney *U* test and comparison between more than two groups was made with the Kruskal-Wallis analysis of variance. Categorical variables were compared with the Chi-square test and Spearman's rank correlation coefficient was used to examine the association of two variables. To assess the main determinants of OSA, we performed binary logistic regression with OSA as a dichotomous (coded as present or absent) dependent variable. As leptin and adiponectin showed associations with age, gender, and BMI, confounding was assessed by including them in the regression model.

## 3. Results

### 3.1. General Results

28.6% of subjects had normal polysomnography, 34.8% had mild OSA, 19.6% had moderate OSA, and 17% had severe OSA. Tables 1 and 2 summarize the clinical, respiratory, and metabolic parameters in the study subjects grouped according to OSA diagnosis and severity. The subjects with severe OSA were found to have larger neck than subjects without OSA and the modified Mallampati score (MMP) correlated with the OSA severity (Table 1). However oropharyngeal crowding and neck circumference showed poor correlations with BMI (*r* = 0.15 (*P* = 0.11) and *r* = 0.13 (*P* = 0.13)), respectively.

**Figure 1:**
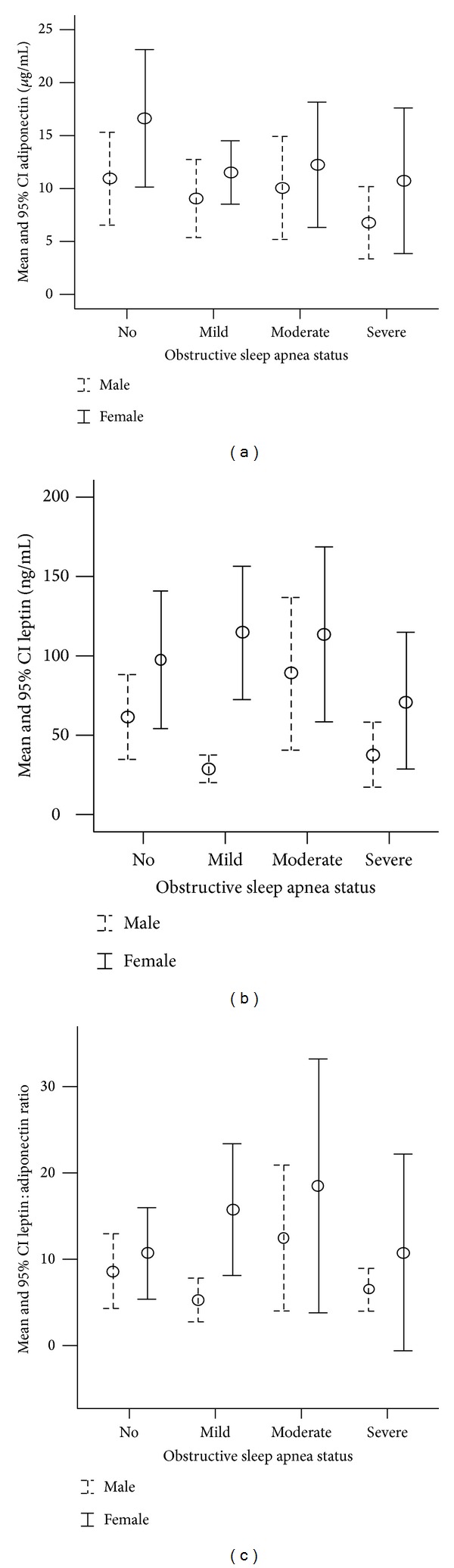
Error-bar (mean and 95% confidence interval) graphs showing gender dimorphism in the association of adipokines with severity of obstructive sleep apnea (OSA).

The metabolic profiles of the three groups are compared in Table 2. Adiponectin decreased significantly (*P* = 0.041) with increasing severity of OSA. However, although BMI was significantly higher in subjects with severe OSA compared to subjects without OSA, leptin was paradoxically lowest in subjects with severe OSA compared to the other groups. Leptin : adiponectin ratio showed variable (significant and nonsignificant) associations with OSA severity. However, the adipokines showed gender dimorphism (Figure 1) but the dimorphism did not affect the consistently lower level of leptin observed in subjects with severe OSA. Total (*P* = 0.019) and LDL-cholesterol levels (*P* = 0.022) increased with increasing OSA severity. Although HDL-cholesterol decreased with increasing OSA severity, the differences were not statistically significant (*P* = 0.74).

### 3.2. Correlations of Adipokines and Clinical and Respiratory Parameters

Table 3 shows the correlations of adiponectin, leptin, and leptin : adiponectin ratio with clinical anthropometric and PSG parameters. Adiponectin showed significant inverse correlation with LDL cholesterol (*r* = −0.21, *P* = 0.026); otherwise, leptin and adiponectin did show significant correlations with lipid and other metabolic parameters (data not shown).

### 3.3. Regression Analyses

The results of binary logistic regression analysis are shown in Table 4. Obesity, as determined by BMI and waist circumference, was not significantly associated with OSA. However MMP and neck circumference were significantly associated with OSA but only MMP was independently associated in the presence of confounding variables. Adiponectin is the only adipokine independently associated with OSA.

## 4. Discussion

Several studies have shown that obesity and OSA share several metabolic and pathophysiologic processes. Studies from different populations have shown that indexes of obesity are predictive factors for OSA [8–11] and as adipocytes are the most important source of adiponectin and leptin, it is expected that circulating leptin and adiponectin would be predictive of OSA severity. Our study shows that lower levels of the protective adipokine and adiponectin are independently associated with OSA and could be used as a marker of disease severity. We have also shown that, paradoxically, subjects with severe OSA have lower leptin. Contrary to expectations, leptin : adeponectin ratio was not independently associated with OSA.

Despite the lack of independent association with OSA on logistic regression analysis, our study suggests that the presence of low levels of leptin in subjects who are obese could be an indicator of the occurrence of severe OSA. A study on a mouse model of obesity showed that leptin could prevent respiratory depression suggesting that lower leptin levels or activity may induce hypoventilation in some obese subjects [31]. Conversely, increase in circulating leptin in subjects with OSA could be a homeostatic response to the hypoventilation and impairment of this response could be the cause of severe OSA. This postulate is supported by the publication of Polotsky et al. [32] who showed that the response to hypoxia was only evident in the obese leptin-deficient ob/ob mice, implying that disruption of leptin pathways may be important for the severity of OSA. However, a cross-sectional study like ours cannot be used to confirm cause-effect relationship between impaired leptin response and OSA severity. Nevertheless, impaired secretion of leptin could also account for the lack of independent association of the leptin : adiponectin ratio with OSA in this study.

Unlike leptin, adiponectin is a protective hormone whose serum levels are inversely related to obesity and insulin resistance [17, 33]. While the effect of hypoxia is known to induce compensatory increase in circulating leptin, less well known is the effect of OSA on adiponectin. In agreement with previous studies [21, 23], we have found lower adiponectin in association with OSA severity suggesting that it could be a good disease marker. Hypoxia has been shown to reduce adiponectin levels via disruption of mechanisms that regulate the production and secretion of adiponectin [34, 35]. Our finding of the lowest adiponectin levels in those with severe OSA is consistent with a potential role for hypoxia as a cause of low adiponectin levels in subjects with OSA. Nevertheless, other factors such as insulin resistance and hypoxia-induced sympathetic activation may also play significant roles [36, 37]. With regard to insulin resistance, our results are in agreement with previous reports [22, 23, 38] that showed that the association between insulin resistance and OSA could be explained by differences in BMI and other confounding variables (Table 4). However, it is pertinent to noting that insulin resistance is widely prevalent in our subjects as the HOMA-IR results (Table 2) are much higher than reported in previous studies from the same population [29].

In this study, we have also shown that increasing oropharyngeal crowding and larger neck circumference (Tables 1 and 4), probably reflecting greater fat or soft tissue fat deposition, are more significantly associated with OSA than traditional indices of obesity. This is an expected finding as increased abdominal obesity could result in reduction in lung volumes and increased fat deposition around the neck and submental region could result in collapse of the upper airway on lying supine.

The lack of a control group of nonobese subjects without OSA could be construed as a weakness in the present study. However, performance of polysomnographic studies on such subjects would not have been clinically indicated or economically feasible. An important strength of this study is that we have studied obese subjects with and without OSA who were similar with respect to various anthropometric and metabolic characteristics. Therefore, any differences cannot be attributed to the expected differences between obese and nonobese subjects.

In conclusion, this study has shown that adiponectin could be a useful marker of disease severity in patients with OSA. Our finding that a paradoxical decrease in circulating leptin occurs in patients with severe OSA deserves further studies in experimental and human models of obesity.

## Figures and Tables

**Table 1:** Clinical and anthropometric characteristics of the study subjects grouped by polysomnographic diagnosis of OSA and OSA severity. Results are mean (standard deviation).

	No	Mild	Moderate	Severe
Age (years)	55.8 (16.3)	49.1 (17.0)	53.0 (16.3)	50.0 (14.8)
Height (m)	1.62 (0.14)	1.62 (0.10)	1.59 (0.12)	1.63 (0.18)
BMI (Kg/m^2^)	40.5 (11.9)	43.3 (10.5)	49.8 (13.9)	44.4 (16.8)
Neck size (cm)	42.8 (5.2)	42.2 (7.5)	44.6 (4.7)	44.4 (3.5)
MMP	2.33 (1.19)	2.59 (1.37)	2.70 (1.17)	2.95 (1.10)*
Saturation of O_2_ on air (%)	94.82 (2.62)	94.36 (4.32)	93.33 (3.45)	95.42 (2.34)
Epwoth's scale	7.69 (5.48)	9.60 (5.39)	10.89 (7.80)	12.44 (7.28)*
Sleep efficiency (%)	83.45 (7.84)	81.44 (7.79)	82.25 (10.77)	81.08 (6.92)
Desaturation index	10.00 (7.45)	20.06 (19.12)	30.38 (20.44)	49.11 (23.20)*
Total number of arousal/hr	95.43 (67.09)	72.73 (46.87)	77.24 (62.37)	69.53 (55.18)
Arousal index/hour	18.39 (12.48)	14.39 (9.16)	16.04 (12.65)	15.61 (13.89)
Basal O_2_ during sleep (%)	92.57 (3.05)	91.90 (3.71)	90.18 (4.89)	90.91 (4.7)
Average low O_2_ during all desaturation (%)	88.036 (3.75)	87.31 (4.24)	83.75 (6.18)	83.92 (5.94)
Minimum O_2_ value (%)	65.49 (21.65)	67.68 (12.87)	57.90 (15.32)	59.65 (13.67)
Basal heart rate (bpm)	72.30 (8.62)	73.57 (10.72)	79.37 (9.59)	75.32 (11.06)
Slowest heart rate (bpm)	44.48 (8.40)	40.23 (8.39)	45.21 (9.02)	44.41 (11.61)
Fastest heart rate (bpm)	100.69 (12.46)	101.51 (11.50)	102.08 (11.12)	104.61
Respiratory disturbance index	2.6 (1.6)	10.3 (3.8)	18.0 (6.9)	48.9 (13.1)*
Hb (g/L)	136.26 (19.17)	130.38 (18.95)	130.88 (25.30)	145.47 (18.53)
WBC (×10^9^/L)	8.16 (3.39)	8.75 (2.57)	9.73 (1.71)	8.80 (4.01)

*P for trend <0.05.

MMP: modified Mallampati score, BMI: body mass index, bpm: beats per minute, Hb: hemoglobin, O_2_: oxygen, and WBC: white blood cells.

**Table 2:** Metabolic characteristics of the study subjects grouped by polysomnographic diagnosis of OSA and OSA severity. Results are mean (standard deviation).

	No	Mild	Moderate	Severe
Adiponectin (ug/mL)	14.70 (13.01)	10.53 (7.41)	10.64 (8.36)	8.87 (7.41)*
Leptin (ug/mL)	70.60 (64.80)	90.90 (86.0)	112.00 (78.60)	52.40 (47.40)
Leptin : adiponectin ratio	7.71 (6.64)	12.80 (15.41)	17.82 (18.24)	8.2 (8.81)
FBG (mmol/L)	6.34 (2.08)	6.27 (1.95)	9.99 (3.66)	8.17 (3.83)
Insulin (mIU/mL)	18.65 (13.51)	20.13 (23.55)	18.67 (13.09)	16.38 (11.05)
HOMA-IR	5.65 (3.98)	5.76 (6.52)	7.66 (6.36)	7.30 (4.54)
*B*%	144.47 (121.38)	118.03 (89.75)	114.94 (30.69)	113.07 (76.51)*
*S*%	61.46 (53.44)	73.83 (51.21)	53.37 (23.79)	42.40 (21.51)
Total cholesterol (mmol/L)	4.30 (1.19)	4.50 (1.02)	4.84 (1.15)	5.07 (1.37)
HDL-C (mmol/L)	1.04 (0.33)	1.08 (0.39)	1.03 (0.39)	0.96 (0.29)
L-CDL (mmol/L)	2.62 (1.04)	2.80 (0.98)	2.90 (0.93)	3.35 (1.22)*
TG (mmol/L)	1.48 (0.77)	1.53 (0.92)	1.77 (1.15)	1.72 (0.85)*

*P for trend <0.05.

FBG: fasting blood glucose, HOMA-IR: homeostasis model assessment of insulin resistance, *B*%: beta cell function, S%: insulin sensitivity, HDL-C: high density lipoprotein cholesterol, LDL-C: low density lipoprotein cholesterol, and TG: triglycerides.

**Table 3:** Correlations of adiponectin, leptin, and leptin : adiponectin ratio with clinical parameters and polysomnographic variables.

Variable	Adiponectin (ng/mL)	Leptin	Leptin : adiponectin ratio
Adiponectin (ng/mL)		0.18** **(0.042)**	−0.50** **(<0.0001)**
Leptin (ng/mL)	0.18** **(0.042)**		0.72** **(<0.0001)**
Leptin : adiponectin ratio	−0.50** **(<0.0001)**	0.72** **(<0.0001)**	
Age (years)	0.34** **(<0.0001)**	0.15 (0.090)	−0.10 (0.269)
BMI (Kg/m^2^)	−0.005 (0.960)	0.54** **(<0.0001)**	0.43** **(<0.0001)**
Height (m)	−0.23** **(0.007)**	−0.30** **(0.001)**	−0.13 (0.157)
Waist (cms)	0.06 (0.539)	0.38** **(<0.001)**	0.23** **(0.017)**
Neck (cms)	−0.19** **(0.025)**	0.05 (0.613)	0.11 (0.216)
MMP	−0.02 (0.858)	−0.07 (0.425)	−0.09 (0.346)
SBP (mm/Hg)	−0.07 (0.461)	0.11 (0.217)	0.12 (0.186)
DBP (mm/Hg)	−0.02 (0.838)	−0.03 (0.757)	0.03 (0.782)
Saturation of O_2_ on air (%)	−0.23** **(0.010)**	−0.28** **(0.002)**	−0.06 (0.531)
Total number of arousal	0.19** **(0.030)**	0.08 (0.392)	−0.10 (0.299)
Arousal index/hour	0.17** **(0.046)**	0.12 (0.202)	−0.06 (0.534)
Basal O_2_ during sleep (%)	−0.06 (0.529)	−0.13 (0.167)	−0.10 (0.298)
Average low O_2_ during all desaturations	−0.09 (0.303)	−0.10 (0.263)	−0.05 (0.589)
minimum O_2_ value	0.05 (0.584)	−0.11 (0.209)	−0.14 (0.130)
RDI	−0.20** **(0.018)**	−0.09 (0.340)	0.05 (0.617)
Basal heart rate (bpm)	0.14 (0.133)	0.26** **(0.005)**	0.13 (0.171)
Slowest heart rate (bpm)	0.04 (0.659)	0.04 (0.676)	0.03 (0.763)
Fastest heart rate (bpm)	0.03 (0.737)	0.15 (0.109)	0.09 (0.343)

Significant correlations are indicated by ∗∗ and BOLD type.

BMI: body mass index, MMP: modified Mallampati score, SBP: systolic blood pressure, DBP: diastolic blood pressure, RDI: respiratory disturbance index.

**Table 4:** Binary logistic regression of the determinants of obstructive sleep apnea in the study subjects.

Variable	Crude odds ratio	*P*	Odds ratio after inclusion of age, sex, and BMI	*P*
Adiponectin (ng/mL)	0.947 (0.898–0.997)	0.039	0.925 (0.871–0.983)	0.012
Leptin (ng/mL)	0.998 (0.990–1.006)	0.589	0.994 (0.985–1.004)	0.225
Leptin : adiponectin ratio	0.993 (0.948–1.041)	0.772	0.982 (0.933–1.032)	0.468
BMI (Kg/m^2^)	1.034 (0.990–1.081)	0.131	1.035 (0.989–1.083)*	0.137
Waist circumference (cm)	1.005 (0.983–1.028)	0.679	1.005 (0.983–1.028)*	0.645
MMP	1.471 (1.087–1.855)	0.022	1.36 (1.12–1.869)	0.031
Neck size (cm)	1.13 (1.004–1.240)	0.042	0.997 (0.933–1.065)	0.113
HOMA-IR	1.16 (1.013–1.307)	0.03	1.024 (0.936–1.120)	0.607

*Only age and sex included in model.

BMI: body mass index, MMP: modified Mallampati score, HOMA-IR: homeostasis model assessment of insulin resistance.
